# Unsteady micropolar nanofluid flow past a variable riga stretchable surface with variable thermal conductivity

**DOI:** 10.1016/j.heliyon.2023.e23590

**Published:** 2023-12-12

**Authors:** Nadeem Abbas, Mohsin Ali, Wasfi Shatanawi, Fady Hasan

**Affiliations:** a Department of Mathematics and Sciences, College of Humanities and Sciences, Prince Sultan University, Riyadh, 11586, Saudi Arabia; b Department of Mathematics, Riphah International University, Faisalabad Campus, Faisalabad, Pakistan; c Department of Medical Research, China Medical University Hospital, China Medical University, Taichung, 40402, Taiwan; d Department of Mathematics, Faculty of Science, The Hashemite University, P.O Box 330127, Zarqa, 13133, Jordan

**Keywords:** Micropolar nanofluid, Riga plate, Nonlinear stretching surface, Variable thermal conductivity

## Abstract

In this study, we considered the flow of a micropolar fluid over a vertical Riga sheet. The non linear stretching sheet is considered. The effects of variable thermal conductivity and radiation on the Riga sheet are taken into account. Additionally, we also debated the Brownian motion and thermophoretic. To simplify the partial differential equations, we converted them into dimensionless ordinary differential equations using suitable similarity variables and solved dimensionless system numerically using the bvp4c function. The impact of some intended parameters on the dimensionless velocity, microrotation, temperature, and concentration distributions graphically are presented and the numerical outcomes of physical quantities like skin friction, Nusselt number, Sherwood number, and couple stress have been presented in tabular form. The micropolar parameter increased which increased the couple stress and friction at surface. Because, the fluid rotation increased which increased friction at surface and also increased the couple stress. The transfer of mass decayed and transfer of heat heightened by larger values of variable thermal conductivity. Thermal conductivity improved which improved the heat transfer phenomena, so transfer of heat at surface becomes larger while also reducing the transfer of mass.

## Nomenclature

(*x, y*)(*m*): Cartesian coordinates
${\;u}_{w}$ (m/s): Velocity
α ($m^{2}/s$): Thermal diffusivity
c (m): Constant
(*u, v*)(m/s): Velocity components
t (s): Time
*g*($m/s^{2}$): Acceleration due to gravity
Q: Heat generation/absorption coefficient
$K_{1}\left(s/m\right)$: Velocity slip factor
$K_{2}$: Thermal slip factor
$K_{3}$: Concentration slip factor
$T_{w}$ (*K*): Free stream temperature
$C_{w}$ (*mol*/ $m^{3}$): Free stream concentration
$\beta_{T\;}$: Thermal expansion coefficient
$\beta_{C\;}$: Concentration expansion coefficient
ν ($m^{2}/s$): Kinematic viscosity
***ρ***($kg/m^{3}$): Fluid density
$k^{*}\left(Ns/m^{2}\right)$: vortex viscosity
k: Thermal conductivity
$\gamma^{*}\left(Ns/m^{2}\right)$: Spin gradient viscosity
*j*$\left(kgm^{2}\right)$: Microinertia per unit mass
*N*: Microrotation
n (1): Constant
***τ***(J/kgK): Heat capacitance and base fluid ratio
$D_{B}$ ($m^{2}/s$): Brownian diffusivity
$C_{p}$ (J/kgK): Specific heat capacitance
$j_{0}$ ($A/m^{2}$): Current density occupied by electrodes
$M_{0}$ (A/m): Magnetization of Riga surface
$q_{r}\left(W/m^{2}\right)$: Rosseland approximation (radiation flux)
${Gr}_{x}$: Thermal buoyancy parameter
θ (1): Temperature
φ (1): Concentration
h (1): Microrotation
***β***(1): Unsteadiness parameter
Pr (1): Prandtl number
*Ec*(1): Eckert number
K(1): Material parameter
Sc(1): Schmidt number
$\beta_{1}\left(1\right)$: Modified Hartmann number
δ: Heat generation parameter
‘: Derivatives with respect to η
ω(1): Buoyancy force parameter
ε(1): Variable thermal conductivity parameter
λ(1): Mixed convection parameter
Nr(1): Radiation parameter
$\delta_{1}$ (1): Velocity slip
$\delta_{2}$ (1): Thermal slip
$\delta_{3}$ (1): Concentration slip
Sh (1): Sherwood number
Nu(1): Nusselt number
$C_{m}\left(1\right)$: Couple stress
$R_{e}$ (1): Local Reynold number
${\;\delta }_{1}\left(1\right)$: Thermal relaxation parameter
${\;\delta }_{2}\left(1\right)$: Concentration relaxation parameter
*e*(1): Exponential
T (K): Prescribed temperature
C (*mol*/ $m^{3}$): Prescribed concentration
${\;T}_{0}$ (K): Reference temperature
$C_{0}$ (*mol*/ $m^{3}$): Reference concentration
${{Gr}_{x}}^{*}$: Concentration buoyancy parameter

## 1 Introduction

Many industrial processes rely on the creation of a magnetic field, and electromagnetic forces are used to control the movement of fluids. In the classical Magnetohydrodynamics (MHD) system, fluid transportation is organized when a large electrically conducting fluid is involved in the process. However, when less-conducting fluids, such as electrolytes, plasma, and liquid metals, are used, the induced currents are very small, and an external magnetic field is required to efficiently control fluid flow. This is the best way to overcome the poor conductivity of the fluids. The crossing of electric and magnetic fields generates the Lorentz force in the direction of the wall, which is an efficient way to intensify the poorly conducting fluids. This mechanism is known as a Riga plate, introduced by Gailitis and Lielausis [1]. The Riga plate is designed in such a way that permanent magnets and alternating arrangement of electrodes align in a span-wise order and extend over a plane surface. The momentum equation is modified, and the Grinberg term [2] is incorporated instead of the Hartmann term to completely decouple the Lorentz force directed to the wall. This term is usually reduced to the plate in exponential form. The Riga surface has gained significant attention and has become an active area of current research. Ahmed et al. [3] have illustrated the transport of nanofluids over a Riga plate by considering zero mass flux. Iqbal et al. [4] have discussed the bio-convective nanofluid dynamics over a variably thick Riga plate by assuming heat and mass fluxes. The investigation of radiative travel of fluid, taking convective heat characteristics into account, produced by a vertical Riga plate was carried out by Nayak et al. [5]. Ramzan et al. [6] have depicted the Williamson nanofluid transportation by assuming a Riga surface with variable thickness under the action of a heat source/sink. For more details, one can refer to Refs. [7,8]. Nanofluids have a wide range of applications in engineering processes that require efficient heat transport. These include large energy devices, automotive cooling, aircraft, space applications, and heat exchangers. According to Choi [9], nanofluids are a special kind of fluids prepared by mixing nano-sized particles in a host or carrier fluid. Traditional fluids have limited applications in high-energy processes and are unsuitable for improving thermal conductivity. Nanofluids, on the other hand, are highly effective in enhancing thermal conductivity due to their large thermal conductivity. Jang and Choi [10] have highlighted that nanofluid thermal conductivity can be further improved by Brownian motion. Buingirno [11] argues that thermophoresis also plays a crucial role in improving the thermal properties of nanofluid. However, this occurs more effectively in convective states than in thermophysical properties. For more information, please refer to Refs. [[12], [13], [14], [15], [16]]. Stretching surfaces play a crucial role in several engineering applications where heat transfer rates and velocity gradients directly affect the final product. The polymer industry, for instance, involves processes such as the extrusion of plastic sheets, drawing wires, glass blowing, crystal growth, heat reduction of continuous filaments, and paper production, all of which require the stretching of surfaces. In most cases, the velocity of the stretched surface is nonlinear, possibly exponential or nonlinear, depending on the situation. Sakiadis [17] conducted revolutionary work on fluid transport on a stretchable surface with continuous motion and constant speed. Crane [18] conducted steady flow analysis on a linearly stretchable plate. Gupta and Gupta [19] adopted the same approach and highlighted suction and blowing cases. Carragher and Crane [20] analyzed the flow through a sheet in a continuous stretching manner, while Magyari and Keller [21] addressed the boundary layer transportation of fluid on an exponentially stretchable surface. Other significant attempts are listed in sections [22,23]. Micropolar fluids are non-Newtonian fluids with microstructure where micro-rotation becomes important. These fluids have recently gained a lot of interest due to their substantial role in various industrial and engineering fields. They have important applications in liquid crystals, colloidal and polymeric suspensions, blood, turbulent shear flows, engine lubricants, and paints. The introduction of micropolar fluid was pioneered by Eringen [24], and researchers have conducted wide exploration with various geometrical approaches. The boundary layer transport of micropolar fluid was deliberated by Peddieson and McNitt [25], and Sankara and Watson [26] presented flow through a stretchable sheet for micropolar fluid. Various studies can be seen in Refs. [[27], [28], [29], [30], [31], [32]]. In our research, we investigated the flow of a micropolar fluid over a stretchable vertically Riga surface. We also looked at how thermal conductivity and radiation affect the fluid. Additionally, we studied how the movement of nanoparticles affects the fluid, taking into account thermophoretic and Brownian motion. To simplify the equations, we converted them into dimensionless ordinary (ODE's) forms using similarity variables. We used the bvp4c function to find a numerical solution. Our results show how physical parameters such as velocity, temperature, concentration, micropolar distribution, Nusselt number, Sharewood number, skin friction, and couple stress are affected by these factors. We presented our findings in graphical and tabular form, which can be applied to industrial and engineering problems. The present model will be extended for the non-Newtonian fluid model using the micropolar fluid model.

## 2 Formulation of problem

**Fig. 1:**
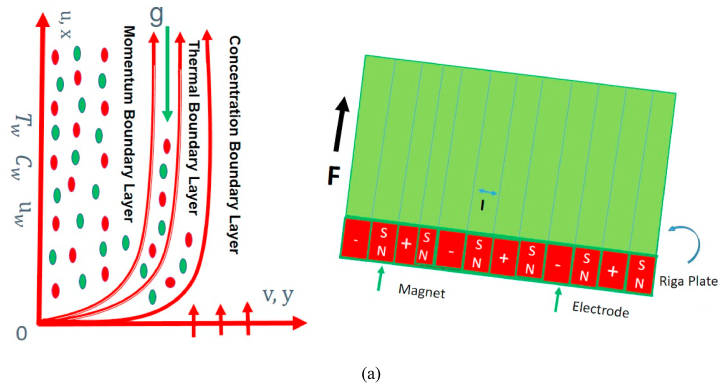

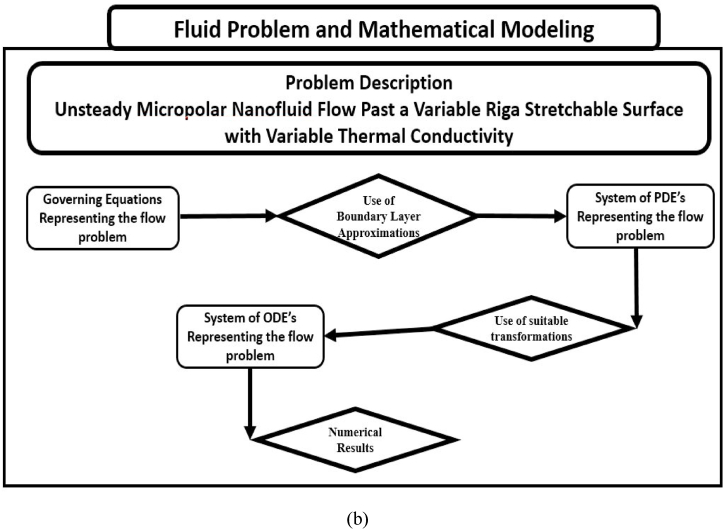

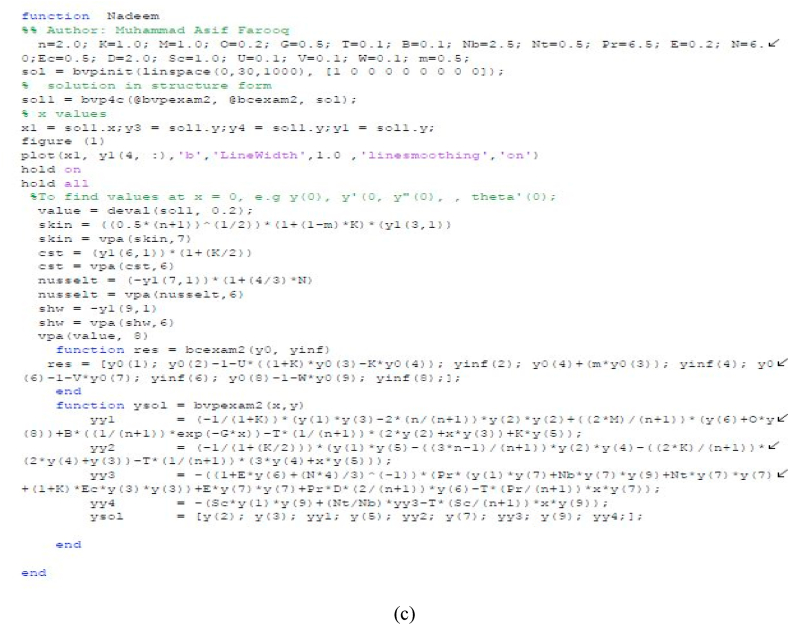
A:The illustration of flow geometry on Riga surface. B:Working Flow diagram. C:Working bvp4c in matlab software packages.

Two dimensional flow of unsteady micropolar nanofluid over a nonlinear stretching vertical Riga sheet. The length l is supposed in such a way that the sheet is stretched along the x-axis with a velocity in the form of $u_{w}=\frac{cx^{n}}{\left(1-\alpha t\right)}$, c > 0 where c is constant. The flow pattern is defined in Fig. 1. The thermal conductivity is taken to be variable. Furthermore, $T_{w}$ and $C_{w}$ are the temperature and concentration at surface, whereas $T_{\infty }$ and ${\;C}_{\infty }$ are assumed to be temperature and concentration at an ambient state far away from the assumed surface respectively. The assumptions of the flow problem is as following:

• Unsteady micropolar fluid flow
• Buongiorno model
• Thermal, velocity and concentration slip
• Nonlinear vertical stretching Riga sheet
• Radiation and variable thermal conductivity
• Viscous dissipation

The leading expressions for flow equations are composed as (see Refs. [[25], [26], [27], [28], [33]]):

(1) $$\frac{\partial u}{\partial x}+\frac{\partial v}{\partial y}=0\text{,}$$

(2) $$\frac{\partial u}{\partial t}+u\frac{\partial u}{\partial x}+v\frac{\partial u}{\partial y}=\nu \left(1+\frac{k^{*}}{\mu }\right)\frac{\partial^{2}u}{\partial y^{2}}+\frac{k^{*}}{\rho }\frac{\partial N}{\partial y}+g\beta_{T}\left(T-T_{\infty }\right)+g\beta_{C}\left(C-C_{\infty }\right)+\frac{M_{o}j_{o}}{8\pi }e^{-\frac{\pi }{a}y}\text{,}$$

(3) $$\frac{\partial N}{\partial t}+u\frac{\partial N}{\partial x}+v\frac{\partial N}{\partial y}=\frac{\gamma }{j\rho }\left(\frac{\partial^{2}N}{\partial y^{2}}\right)-\frac{k^{*}}{j\rho }\left(2N+\frac{\partial u}{\partial y}\right)\text{,}$$

(4) $$\frac{\partial T}{\partial t}+u\frac{\partial T}{\partial x}+v\frac{\partial T}{\partial y}=\frac{1}{\rho C_{\rho }}\frac{\partial }{\partial y}\left(k\left(T\right)\frac{\partial T}{\partial y}\right)+\left(\frac{\mu +k^{*}}{\rho C_{\rho }}\right){\left(\frac{\partial u}{\partial y}\right)}^{2}-\frac{1}{\rho C_{\rho }}\frac{\partial q_{r}}{\partial y}+\frac{Q}{\rho C_{\rho }}\left(T-T_{\infty }\right)+\tau \left[D_{B}\frac{\partial C}{\partial y}\frac{\partial T}{\partial y}+\frac{D_{T}}{T_{\infty }}{\left(\frac{\partial T}{\partial y}\right)}^{2}\right]\text{,}$$

(5) $$\frac{\partial C}{\partial t}+u\frac{\partial C}{\partial x}+v\frac{\partial C}{\partial y}=D_{B}\frac{\partial^{2}C}{{\partial y}^{2}}+\frac{D_{T}}{T_{\infty }}\frac{\partial^{2}T}{\partial y^{2}}$$

The subjected boundary conditions are

(6) $$v=0,u=u_{w}+K_{1}\left(\left(\mu +k\right)\frac{\partial u}{\partial y}+kN\right),T=T_{w}+K_{2}\frac{\partial T}{\partial y},C=C_{w}+K_{3}\frac{\partial C}{\partial y},N=-m_{0}\frac{\partial u}{\partial y},as\;y=0\text{,}\;u\to 0,C\to C_{\infty },T\to T_{\infty },N\to 0,at\;y\to \infty \text{.}$$

Here, *u* and *v* are expressing the velocity components along of x and y direction of axes respectively. $K_{1}$, $K_{2}$ and $K_{3}$ are the velocity, thermal and concentration slip factor, respectively. $\beta_{T\;}$ and $\beta_{C}$ both are representing volumetric coefficients of thermal expansion and concentration expansion accordingly. The symbol g is acceleration due to gravity while Q signifies heat generation/absorption constant. The symbol $j_{0}$ is denoting the applied current density occupied by electrodes. The Riga plate has permanent magnets which are mounted on its surface and their magnetization is expressed by symbol $M_{0}$ whereas the width of the magnets inside the electrodes is denoted by a. The other notations are described as density of the fluid ***ρ***, thermal diffusivity ***α***, heat capacitance and base fluid ratio ***τ***, specific heat $C_{p}$, thermophoretic diffusion coefficient $D_{T}$, Brownian diffusivity $D_{B}$, kinematic viscosity ***ν***, and the dynamic viscosity μ respectively. The variable temperature dependent thermal conductivity k(T) can be written as:

(7) $$k\left(T\right)=k_{\infty }\;\left(\;1+\varepsilon \theta \left(\eta \right)\right)\text{.}$$

Where $k_{\infty }$ is thermal conductivity at ambient temperature $T_{\infty }$ and ε is the thermal conductivity parameter. Also the spin gradient viscosity $\gamma^{*}$ in terms of $j=\frac{\nu \left(1-\alpha t\right)}{cx^{n-1}}$ which is the microinertia per unit mass, the material parameter $K=k^{*}/\mu$ which is the ratio of the vortex viscosity ($k^{*}$) to the dynamic viscosity (μ), can be written as:

(8) $$\gamma^{*}=\left(\mu +\frac{k^{*}}{2}\right)j=\mu \left(1+\frac{K}{2}\right)j\text{.}$$

The similarity variables are of the form:

(9) $$u=\frac{cx^{n}}{\left(1-\alpha t\right)}f^{\prime }\left(\;\eta \right),v=-\sqrt{\frac{c\left(n+1\right)}{2\nu \left(1-\alpha t\right)}}x^{\frac{n-1}{2}}\left[f\left(\eta \right)+\frac{n-1}{n+1}\eta f^{\prime }\left(\eta \right)\right],\varphi \left(\;\eta \right)=\frac{C-C_{\infty }}{C_{w}-C_{\infty }}\;\;\eta =\sqrt{\frac{c\left(n+1\right)}{2\nu \left(1-\alpha t\right)}}x^{\frac{n-1}{2}}y,\theta \left(\eta \right)=\frac{T-T_{\infty }}{T_{w}-T_{\infty }},N=\frac{cx^{n}}{\left(1-\alpha t\right)}\sqrt{\frac{c\left(n+1\right)}{2\nu \left(1-\alpha t\right)}}x^{\frac{n-1}{2}}h\left(\eta \right).\;$$

Rosseland approximation (radiation flux) is taken in the form of

(10) $$q_{r}=\frac{-4\sigma }{3k}\frac{{\partial T}^{4}}{\partial y}\text{.}$$

The term $T^{4}$ can be solved in Taylor series about $T_{\infty }$. After ignoring high order expressions, it gives

(11) $$T^{4}\approx 4T_{\infty }^{3}T-3T_{\infty }^{4}\text{.}$$

In view of above equation, we can write

(12) $$\frac{\partial q_{r}}{\partial y}=-\frac{16T_{\infty }^{3}\sigma }{3k}\frac{\partial^{2}T}{\partial y^{2}}$$

The similarity variables defined in set of expressions (9) are identical to satisfy continuity equation (1). After successful utilization of these similarity variables, equations (2−5) take the dimensionless forms as

(13) $$\left(1+K\right)f^{\prime \prime \prime }+{ff}^{\prime \prime }-\left(\frac{2n}{n+1}\right)f^{\prime 2}+\left(\frac{2\lambda }{n+1}\right)\left(\theta +\omega \varphi \right)+\left(\frac{2}{n+1}\right)\beta_{1}e^{-\gamma \eta }-\left(\frac{2}{n+1}\right)\beta \left(f^{\prime }+\frac{\eta }{2}f^{\prime \prime }\right)+Kh^{\prime }=0$$

(14) $$\left(1+\frac{K}{2}\right)h^{\prime \prime }+fh^{\prime }-\left(\frac{3n-1}{n+1}\right)f^{\prime }h-\left(\frac{2K}{n+1}\right)\left(2h+f^{\prime \prime }\right)-\left(\frac{1}{n+1}\right)\beta \left(3h+\eta h^{\prime }\right)=0$$

(15) $$\left(\;1+\varepsilon \theta \right)\theta^{\prime \prime }+{{\frac{4}{3}N}_{r}\theta }^{\prime \prime }+\varepsilon {\theta^{\prime }}^{2}+\mathrm{Pr}\left({f\theta }^{\prime }+Nb\theta^{\prime }\varphi^{\prime }+Nt{\theta^{\prime }}^{2}+Ec\left(1+K\right){f^{\prime \prime }}^{2}\right)+\left(\frac{2}{n+1}\right)\mathrm{Pr}\;\delta \theta -\left(\frac{\mathrm{Pr}}{n+1}\right)\beta \eta \theta^{\prime }=0\text{,}$$

(16) $$\varphi^{\prime \prime }+{Scf\varphi }^{\prime }+\frac{Nt}{Nb}\theta^{\prime \prime }-\left(\frac{Sc}{n+1}\right)\beta \eta \varphi^{\prime }=0\text{.}$$

Where,

(17) $$\mathrm{Pr}=\frac{\nu }{\alpha },\;\beta =\frac{\alpha }{c},\;\nu =\frac{\mu }{\rho },\;K=\frac{k^{*}}{\mu },\;Sc=\frac{\nu }{D_{B}},\;\;Le=\frac{\nu }{D_{B}}\text{.}$$

In the expressions (15), ${Gr}_{x}$ is thermal buoyancy parameter, ${{Gr}_{x}}^{*}$ is concentration buoyancy parameter $\beta_{1}$ is modified Hartmann number, γ is positive constant, ω is showing buoyancy force parameter, ε is parameter of variable thermal conductivity, unsteadiness parameter ***β*** and λ is mixed convection parameter respectively. The symbols Sc Pr, Ec, δ, $N_{r},Nt$ and Nb are denoting Schmidt number, Prandtl number, Eckert number, coefficient of heat generation, radiation parameter, thermophoresis and Brownian movement respectively. The prime ′ on variable quantities is derivatives with respect to η in all equations. The boundary conditions (6) can be expressed as

(18) $$f\left(\eta \right)=0,f^{\prime }\left(\eta \right)=1+\delta_{1}\left({\left(1+K\right)f}^{\prime \prime }\left(\eta \right)+Kh\left(\eta \right)\right),{h\left(\eta \right)=-mf}^{\prime \prime }\left(\eta \right),\theta \left(\eta \right)=1+\delta_{2}\theta^{\prime }\left(\eta \right),\varphi \left(\eta \right)=1+\delta_{3}\varphi^{\prime }\left(\eta \right)\;at\;\eta =0,f^{\prime }\left(\eta \right)\to 0,h\left(\eta \right)\to 0,\theta \left(\eta \right)\to 0,\varphi \left(\eta \right)\to 0\;at\;\eta \to \infty \text{.}$$

Where $\delta_{1}$ is velocity slip, $\delta_{2}$ is heat slip and $\delta_{3}$ is mass slip accordingly. Note that m lies in 0≤m≤1 and m=0 results N=0 near the wall. This is strong concentration (concentrated particle flows) case and the microelements rotations close to the wall can't take place. m=1 lies in turbulent flows cases. In current analysis, only m=0.5 is taken because the antisymmetric part in the stress tensor vanishes when m=0.5 and concentrations of microelements become weak. The working flow diagram is presented by Fig. 1. The skin friction, Nusselt number, Sherwood number and couple stress on the wall are valuable physically important quantities. These quantities relevant to employed model are given by

(19) $$C_{f}=\frac{\tau_{w}}{\rho u_{w}^{2}},{Nu}_{x}=\frac{xq_{w}}{k_{1}\left(T_{f}-T_{\infty }\right)},{Sh}_{x}=\frac{xq_{m}}{D_{B}\left(C_{f}-C_{\infty }\right)},C_{m}=\frac{\tau_{m}}{\mu ju_{w}}\text{.}$$

Where the expressions of shear stress, heat flux, mass flux and wall couple stress the wall accordingly are defined below

(20) $$\tau_{w}=\left[\left(\mu +k^{*}\right)\frac{\partial u}{\partial y}+k_{N}^{*}\right]{|}_{y=0},q_{w}=\left[-k_{1}\left(\frac{\partial T}{\partial y}\right)+q_{r}\right]{|}_{y=0},q_{m}=-D_{B}\left(\frac{\partial C}{\partial y}\right){|}_{y=0},\tau_{m}=\left(\mu +\frac{k^{*}}{2}\right)j\left(\frac{\partial N}{\partial y}\right){|}_{y=0}\text{.}$$

The skin friction, Nusselt number, Sherwood number and couple stress in their relevant non-dimensional appearance in the form of local Reynold number $R_{e}=\frac{u_{w}x}{\nu }$ are displayed as:

(21) $${C_{f}R}_{e}^{1/2}={\sqrt{\frac{n+1}{2}}\left(1+\left(1-m\right)K\right)f}^{\prime \prime }\left(0\right),{NuR}_{e}^{1/2}={-\left(1+{\frac{4}{3}N}_{r}\right)\theta }^{\prime }\left(0\right),ShR_{e}^{1/2}={-\varphi }^{\prime }\left(0\right),{C_{m}R}_{e}^{1/2}=\left(1+\frac{K}{2}\right)h^{\prime }\left(0\right)\text{.}$$

## 3 Numerical procedure

The dimensionless system of differential equations are solved numerical using the matlab software package. Numerical procedure of the differential equations are transformed in the first order differential equations. The selection of finite value is ξ→∞ as ξ=20 which shows that the present results are corrected asymptotically for numerical technique. The $10^{-6}$ is the tolerance error of convergence criteria and process is adopted as following:

(22) $$Y\left(1\right)=f\left(\eta \right);\;Y\left(2\right)=f^{\prime }\left(\eta \right);Y\left(3\right)=f^{\prime \prime }\left(\eta \right);\;YY1=f^{\prime \prime \prime }\left(\eta \right)\text{;}$$

(23) $$YY1=\frac{-1}{\left(1+K\right)}\left(Y\left(1\right)Y\left(3\right)-\left(\frac{2n}{n+1}\right)Y\left(2\right)Y\left(2\right)+\left(\frac{2\lambda }{n+1}\right)\left(Y\left(6\right)+\omega Y\left(8\right)\right)+\left(\frac{1}{n+1}\right)\beta_{1}e^{-\gamma \eta }-\left(\frac{1}{n+1}\right)\beta \left(2Y\left(2\right)+\eta Y\left(3\right)\right)+KY\left(5\right)\right)\text{;}$$

(24) $$Y\left(4\right)=h\left(\eta \right);\;Y\left(5\right)=h^{\prime }\left(\eta \right);\;YY2=h^{\prime \prime }\left(\eta \right)\text{;}$$

(25) $$YY2=\frac{-1}{\left(1+\frac{K}{2}\right)}\left(Y\left(5\right)Y\left(1\right)-\left(\frac{3n-1}{n+1}\right)Y\left(2\right)Y\left(4\right)+\left(\frac{2K}{n+1}\right)\left(2Y\left(4\right)+Y\left(3\right)\right)-\left(\frac{1}{n+1}\right)\beta \left(Y\left(4\right)+\eta Y\left(5\right)\right)\right)\text{;}$$

(26) $$Y\left(6\right)=\theta \left(\eta \right);\;Y\left(7\right)=\theta^{\prime }\left(\eta \right);\;YY3=\theta^{\prime \prime }\left(\eta \right)\text{;}$$

(27) $$YY3=\frac{-1}{\left(\;1+\varepsilon \theta +{\frac{4}{3}N}_{r}\right)}\left(\varepsilon Y\left(7\right)Y\left(7\right)+\mathrm{Pr}\left(Y\left(7\right)Y\left(1\right)+NbY\left(7\right)Y\left(9\right)+NtY\left(7\right)Y\left(7\right)+EcY\left(3\right)Y\left(3\right)\right)+\left(\frac{2}{n+1}\right)\mathrm{Pr}\;\delta Y\left(6\right)-\left(\frac{\mathrm{Pr}}{n+1}\right)\beta \eta Y\left(7\right)\right)\text{;}$$

(28) $$Y\left(8\right)=\varphi \left(\eta \right);\;Y\left(9\right)=\varphi^{\prime }\left(\eta \right);\;YY4=\varphi^{\prime \prime }\left(\eta \right)\text{;}$$

(29) $$YY4=-\left(ScY\left(9\right)Y\left(1\right)+\frac{Nt}{Nb}\text{YY}3-\left(\frac{Sc}{n+1}\right)\beta \eta Y\left(9\right)\right)\text{;}$$

Having the boundary conditions are

(30) $$Y_{0}\left(1\right);\;Y_{0}\left(2\right)-1-\delta_{1}(\left(1+K\right)Y_{0}\left(3\right)+KY_{0}\left(4\right));\;Y_{0}\left(4\right)+mY_{0}\left(3\right);\;Y_{0}\left(6\right)-1-\delta_{2}Y_{0}\left(7\right)\;;\;Y_{0}\left(8\right)-1-\delta_{3}Y_{0}\left(9\right);\;Y_{\infty }\left(2\right);\;Y_{\infty }\left(4\right);\;Y_{\infty }\left(6\right);\;Y_{\infty }\left(8\right)\text{;}$$

Fig. 1 reported the code bvp4c using the matlab software packages in details. Utilizing Newton's method, we refine the initial approximations until we meet the necessary convergence criteria with confidence. See below for the boundary residuals:

$$R_{1}=\left|Y_{2}\left(\infty \right)-\hat{Y_{2}}\left(\infty \right)\right|\text{,}$$

$$R_{2}=\left|Y_{4}\left(\infty \right)-\hat{Y_{4}}\left(\infty \right)\right|\text{,}$$

$$R_{3}=\left|Y_{6}\left(\infty \right)-\hat{Y_{6}}\left(\infty \right)\right|\text{,}$$

$$R_{4}=\left|Y_{8}\left(\infty \right)-\hat{Y_{8}}\left(\infty \right)\right|\text{,}$$

The residuals of microrotation ($R_{2}$), concentration ($R_{4}$), temperature ($R_{3}$) and velocity ($R_{1}$) are presented.

## 4 Results and discussion

**Fig. 2:**
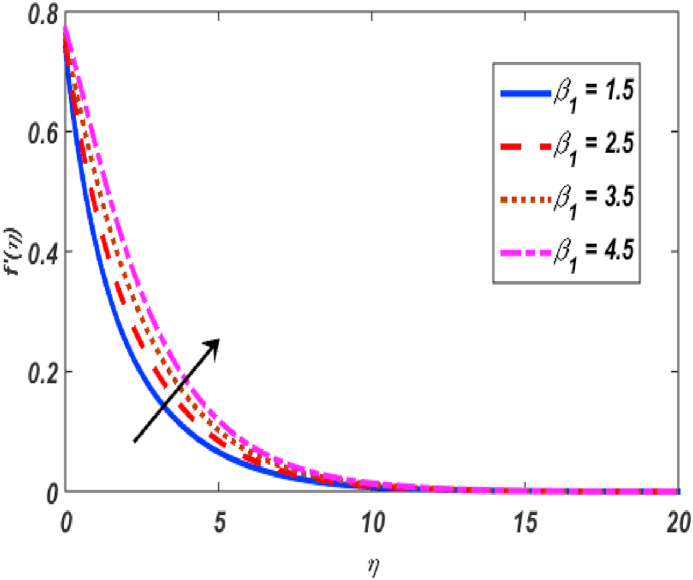
Variation of $f^{\prime }\left(\eta \right)$ for $\beta_{1}$ along η..

**Fig. 3:**
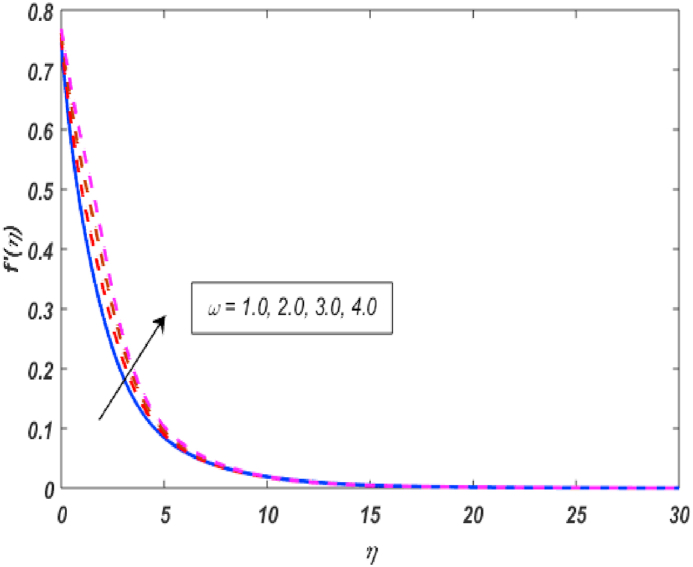
Variation of $f^{\prime }\left(\eta \right)$ for ω along η..

**Fig. 4:**
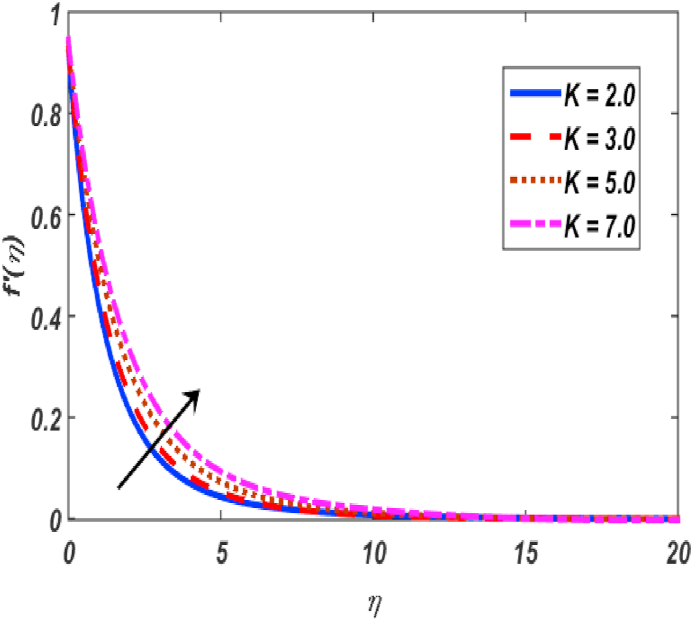
Variation of $f^{\prime }\left(\eta \right)$ for K along η..

**Fig. 5:**
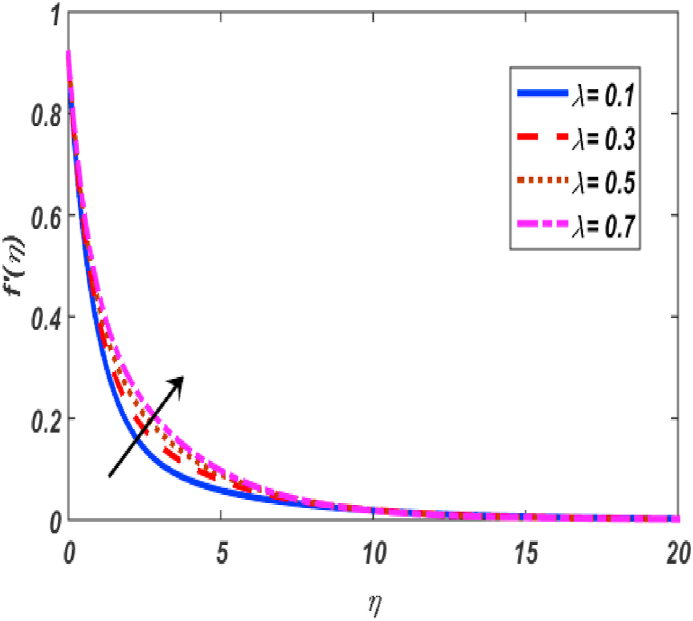
Variation of $f^{\prime }\left(\eta \right)$ for λ along η..

**Fig. 6:**
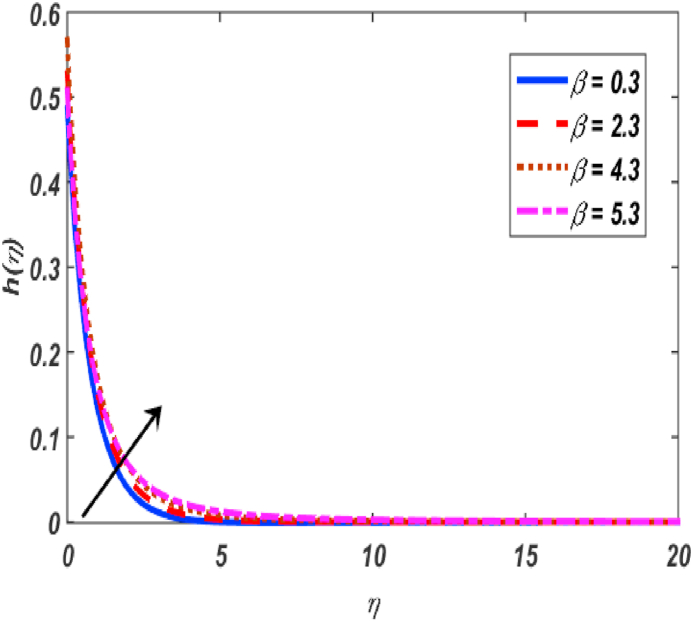
Variation of h(η) for β along η..

**Fig. 7:**
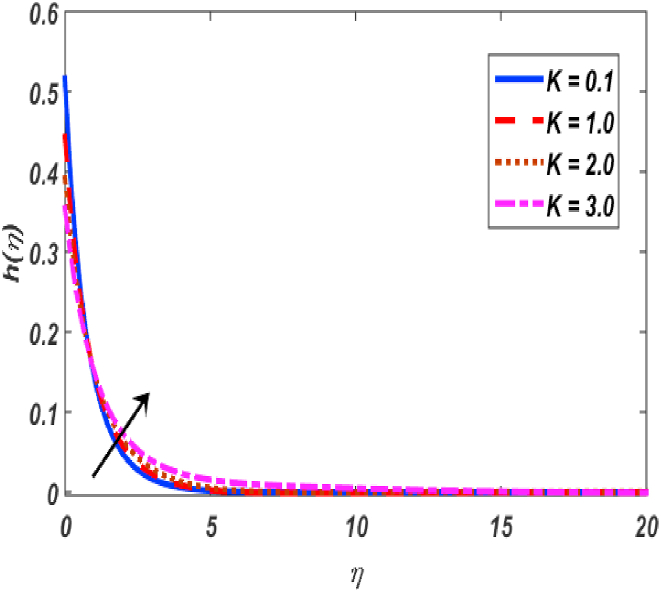
Variation of h(η) for K along η..

**Fig. 8:**
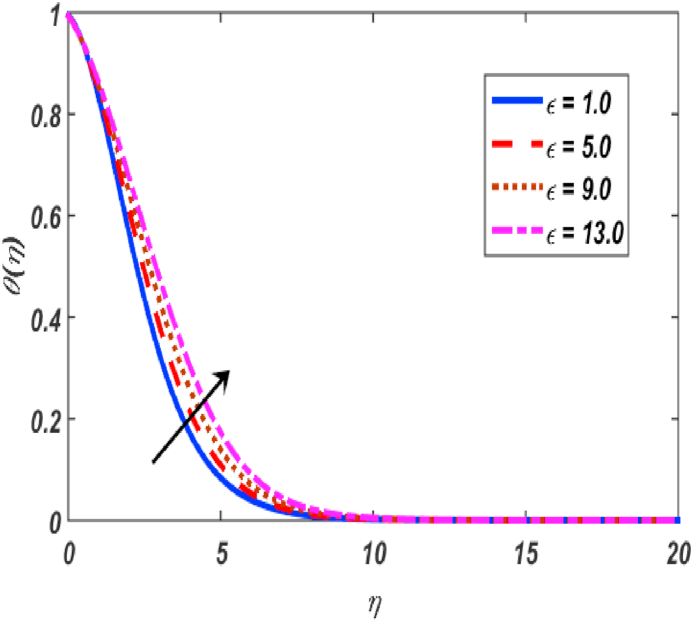
Variation of θ(η) for ε along η..

**Fig. 9:**
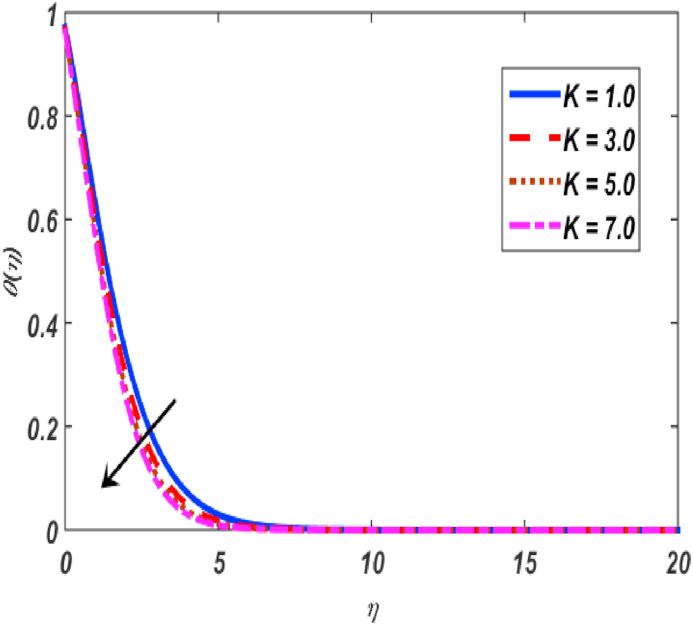
Variation of θ(η) for K along η..

**Fig. 10:**
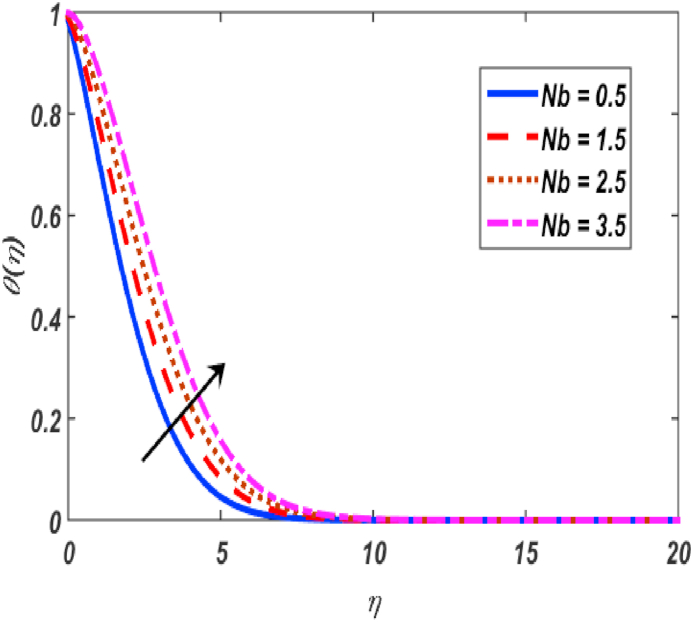
Variation of θ(η) for Nb along η..

**Fig. 11:**
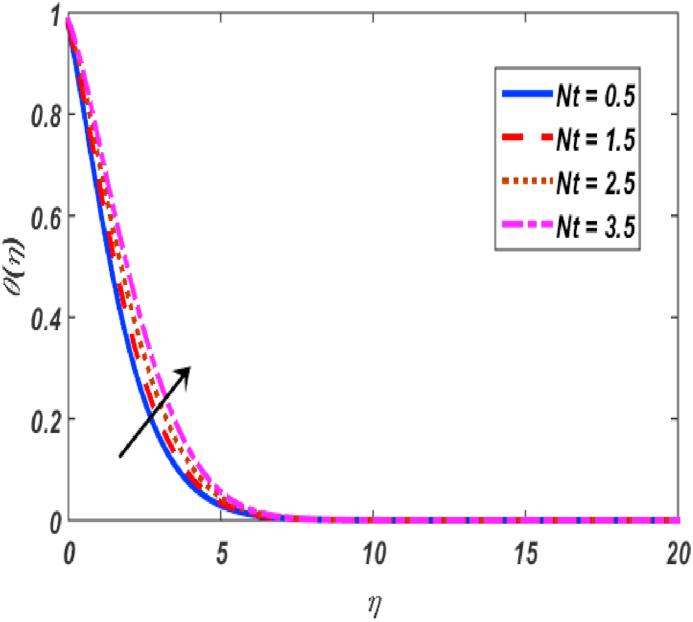
Variation of θ(η) for Nt along η..

**Fig. 12:**
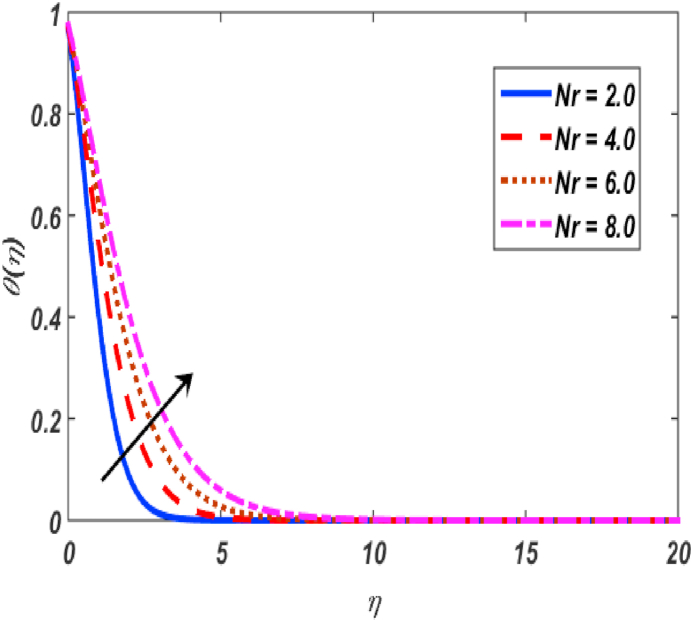
Variation of θ(η) for Nr along η..

**Fig. 13:**
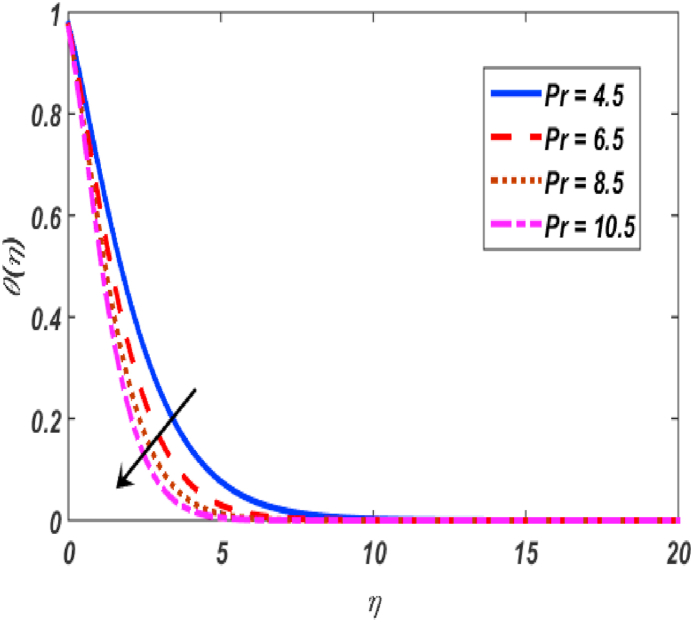
Variation of θ(η) for Pr along η..

**Fig. 14:**
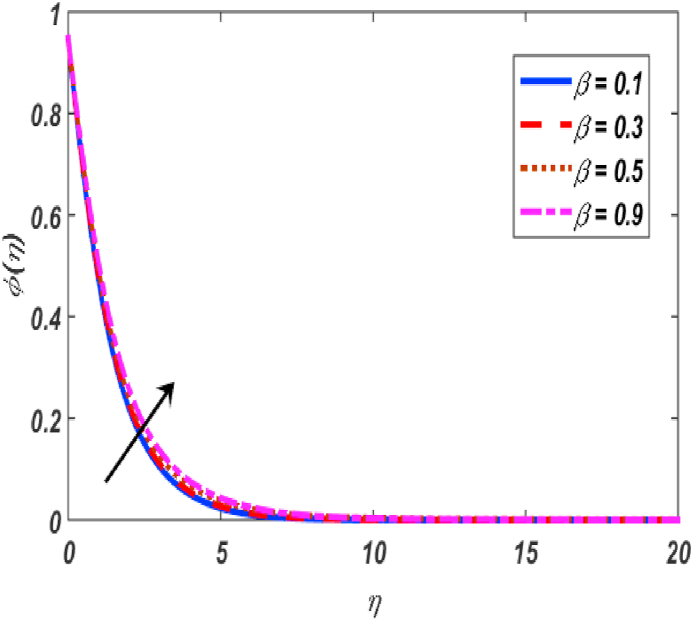
Variation of φ(η) for β along η..

**Fig. 15:**
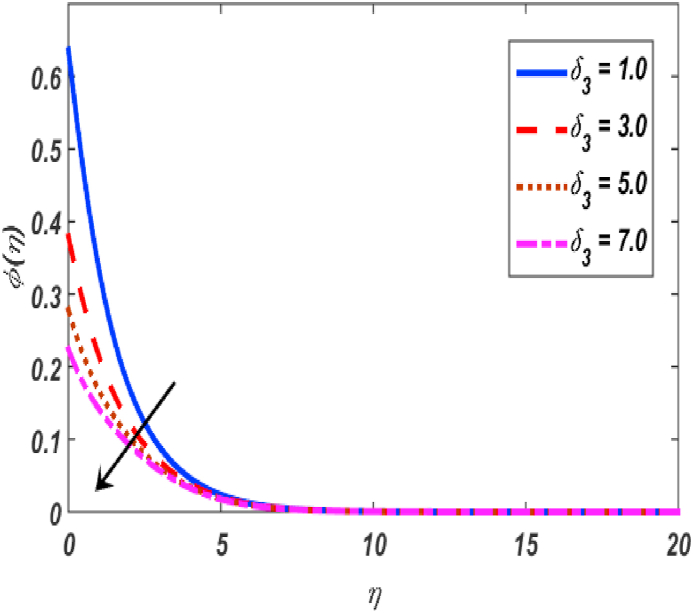
Variation of φ(η) for $\delta_{3}$ along η..

**Fig. 16:**
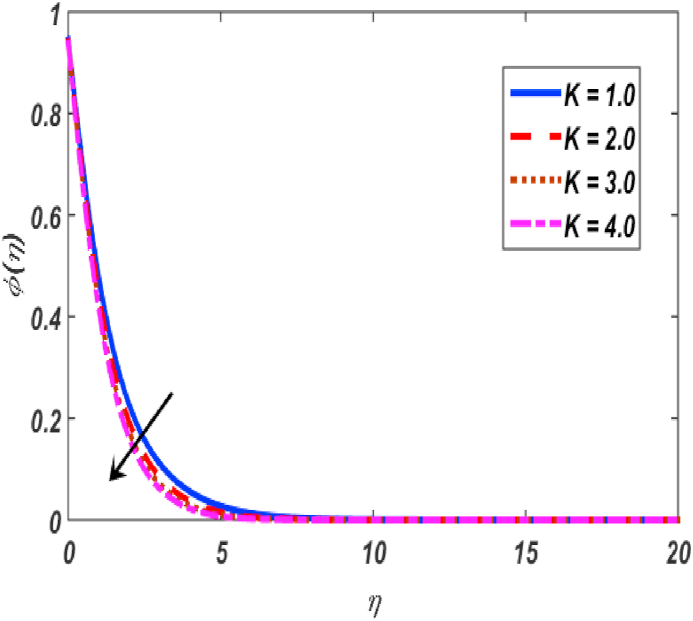
Variation of φ(η) for K along η..

**Fig. 17:**
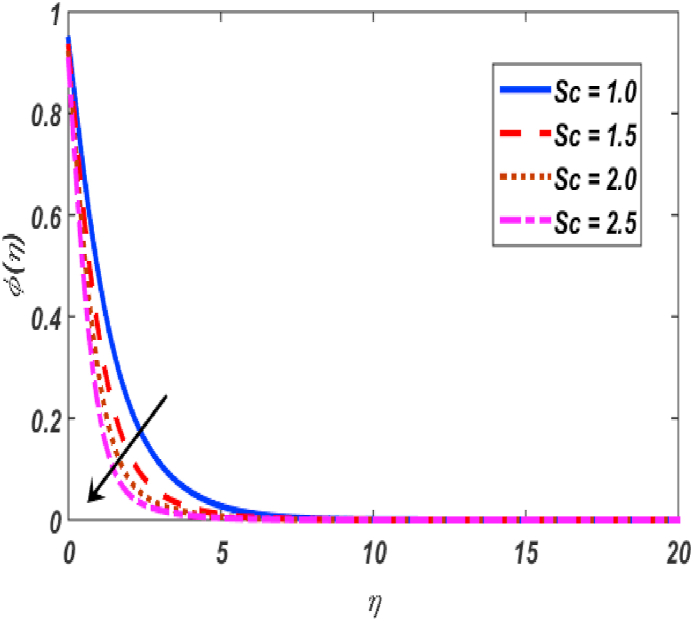
Influence of φ(η) for Sc along η..

Built-in bvp4c method in Matlab software is utilized on the set of expressions (11–14) with conditions (16) to calculate numerical results. The major theme of this section is the computation of numerical achievements for governing parameters which are modified Hartmann number $\beta_{1}$, unsteadiness parameter β, buoyancy force parameter ω, positive constant γ, variable thermal conductivity ε, radiation parameter $N_{r}$, Eckert number Ec, material parameter K, Schmidt number Sc, Prandtl number Pr, heat generation coefficient δ, mixed convection parameter λ and thermophoresis Nt, Brownian movement parameter Nb, velocity slip $\delta_{1}$, thermal slip $\delta_{2}$ and concentartion slip $\delta_{3}$. To visualize the effects of parameters on flow velocity, microrotation, temperature, and concentration profiles, the support of graphical diagrams is taken and described through Fig. 2, Fig. 3, Fig. 4, Fig. 5, Fig. 6, Fig. 7, Fig. 8, Fig. 9, Fig. 10, Fig. 11, Fig. 12, Fig. 13, Fig. 14, Fig. 15, Fig. 16, Fig. 17. Table 1 is prepared to analyze the variations in skin friction (${C_{f}Re}_{s}^{1/2})$ and couple stress (${C_{m}R}_{e}^{1/2})$ whereas Table 2 is prepared to present the variations in Nusselt number (${-\theta }^{\prime }\left(0\right))$ and Sherwood number (${-\varphi }^{\prime }\left(0\right)$) against variations of various physical parameters. The friction at a Riga sheet boosted due to improving values of n. If the values of n improved which improved the shear thickening as well as improved the friction between fluid and surface. The couple stress declined due to higher values of n. If the values of n improved which improved the shear thickening as well as declined the couple stress because viscosity of fluid increased which affected the couple stress at surface. The impact of K on the skin friction and couple's stress noted. The K increased which increased the couple stress and friction at surface. Because, the fluid rotation increased which increased friction at surface and also increased the couple stress. Skin friction boosted up while couple stress declined for higher values of β. Because, the momentum thickness of boundary layer reduced as well as reduced in velocity. So, the friction increased due to reduction in the momentum thickness at surface. The larger values of $\beta_{1}$ which increment in friction and reduction couple stress at surface. The Laurent forces developed the resistance between surface and fluid due to a reduction in the momentum thickness and ultimately, increment in skin friction and couple stress declined. It is manifested that both friction and couple stress are increased by varying γ to bigger values. Physically, the gap between surface and magnetic field declined exponentially which reduced the momentum thickness ultimately, increased the friction at surface. It is manifested that both friction and couple stress are condensed by varying λ to bigger values. The buoyancy force reduced the friction between surface and fluid interaction point. It is manifested that both friction and couple stress are condensed by varying ω to bigger values. It is displayed that both friction and couple stress are condensed by varying ε to bigger values. Thermal conductivity improved which improved the heat transfer phenomena, so friction between surface and fluid become lesser while also reduced the couple stress. The increment in Nb which is reduced the friction while heightening the couple stress. The increment in Nt which reduced the friction while heightened the couple stress. The couple stress values heightened and friction values reduced due to enlarging values of Pr. The diffusion of momentum enlarged due to heightening the values of Pr as well as reduced the friction and increment in couple stress. The friction is decreased whereas couple stress is increased, when values of Ec increased. If the values of Ec boosted up which heightened the kinetic energy as well as increment in momentum. Due to increment in momentum, the friction reduced at surface while couple stress enhanced. The friction is decreased whereas couple stress is increased, when values of δ increased. If the values of δ boosted up which heightened the kinetic energy as well as increment in momentum. Due to increment in momentum, the friction is reduced at surface while couple stress enhanced. The increment in Nr which reduced the friction and the couple stress. The friction is decreased whereas couple stress is increased when values of Sc increased. The heighten values of $\delta_{1}$ which reduced the couple stress and friction at surface. As the slip factor boosted up which reduced the friction between fluid and surface while also reducing the couple stress. The increment in $\delta_{2\;}$ which reduced the friction while heightening the couple stress. The increment in $\delta_{3\;}$ which heightened the friction while reducing the couple stress.

**Table 1:** The numerical calculations computed for the skin friction ${C_{f}Re}_{e}^{1/2}$ and couple stress ${C_{m}R}_{e}^{1/2}$ by setting m=0.5 fixed.

n	K	β	$\beta_{1}$	γ	λ	ω	ε	Nb	Nt	Pr	Ec	δ	Nr	Sc	$\delta_{1}$	$\delta_{2}$	$\delta_{3}$	${C_{f}Re}_{e}^{1/2}$	${C_{m}R}_{e}^{1/2}$
2.0	1.0	0.1	0.1	0.5	0.1	0.2	0.2	2.5	0.5	6.5	0.5	0.1	6.0	1.0	0.1	0.1	0.1	−1.178609	1.50426
3.0	–	–	–	–	–	–	–	–	–	–	–	–	–	–	–	–	–	−1.430443	1.50357
4.0	–	–	–	–	–	–	–	–	–	–	–	–	–	–	–	–	–	−1.643741	1.50315
2.0	1.0	0.1	0.1	0.5	0.1	0.2	0.2	2.5	0.5	6.5	0.5	0.1	6.0	1.0	0.1	0.1	0.1	−1.430443	1.50357
–	2.0	–	–	–	–	–	–	–	–	–	–	–	–	–	–	–	–	−1.254313	2.00661
–	3.0	–	–	–	–	–	–	–	–	–	–	–	–	–	–	–	–	−1.318677	2.50855
2.0	1.0	0.2	0.1	0.5	2.0	0.2	0.2	2.5	0.5	6.5	0.5	0.1	6.0	1.0	0.1	0.1	0.1	−0.3041234	1.48341
–	–	0.3	–	–	–	–	–	–	–	–	–	–	–	–	–	–	–	−0.3233543	1.48511
–	–	0.4	–	–	–	–	–	–	–	–	–	–	–	–	–	–	–	−0.3399724	1.48637
2.0	1.0	0.1	0.1	0.5	0.1	0.2	0.2	2.5	0.5	6.5	0.5	0.1	6.0	1.0	0.1	0.1	0.1	−1.430443	1.50357
–	–	–	0.2	–	–	–	–	–	–	–	–	–	–	–	–	–	–	−1.157339	1.50329
–	–	–	0.3	–	–	–	–	–	–	–	–	–	–	–	–	–	–	−1.136196	1.50235
2.0	1.0	0.1	0.1	0.6	0.1	0.2	0.2	2.5	0.5	6.5	0.5	0.1	6.0	1.0	0.1	0.1	0.1	−1.180456	1.50444
–	–	–	–	0.7	–	–	–	–	–	–	–	–	–	–	–	–	–	−1.181963	1.50457
–	–	–	–	0.8	–	–	–	–	–	–	–	–	–	–	–	–	–	−1.183226	1.50468
2.0	1.0	0.1	0.1	0.5	1.0	0.2	0.2	2.5	0.5	6.5	0.5	0.1	6.0	1.0	0.1	0.1	0.1	−0.7102462	1.49006
–	–	–	–	–	1.5	–	–	–	–	–	–	–	–	–	–	–	–	−0.4907673	1.48642
–	–	–	–	–	2.0	–	–	–	–	–	–	–	–	–	–	–	–	−0.2851682	1.48399
2.0	1.0	0.1	0.1	0.5	1.0	0.2	0.2	2.5	0.5	6.5	0.5	0.1	6.0	1.0	0.1	0.1	0.1	−0.7102462	1.49006
–	–	–	–	–	–	0.4	–	–	–	–	–	–	–	–	–	–	–	−0.6579586	1.48919
–	–	–	–	–	–	0.6	–	–	–	–	–	–	–	–	–	–	–	−0.6059157	1.48864
2.0	1.0	0.1	0.1	0.5	1.0	0.2	0.2	2.5	0.5	6.5	0.5	0.1	6.0	1.0	0.1	0.1	0.1	−0.7102462	1.49006
–	–	–	–	–	–	–	0.4	–	–	–	–	–	–	–	–	–	–	−0.7098547	1.48983
–	–	–	–	–	–	–	0.6	–	–	–	–	–	–	–	–	–	–	−0.7094599	1.48961
2.0	1.0	0.1	0.1	0.5	1.0	0.2	0.2	2.5	0.5	6.5	0.5	0.1	6.0	1.0	0.1	0.1	0.1	−0.7102462	1.49006
–	–	–	–	–	–	–	–	2.6	–	–	–	–	–	–	–	–	–	−0.7084482	1.49072
–	–	–	–	–	–	–	–	2.7	–	–	–	–	–	–	–	–	–	−0.7063916	1.49147
2.0	1.0	0.1	0.1	0.5	1.0	0.2	0.2	2.5	0.5	6.5	0.5	0.1	6.0	1.0	0.1	0.1	0.1	−0.7102462	1.49006
–	–	–	–	–	–	–	–	–	0.6	–	–	–	–	–	–	–	–	−0.7081771	1.49033
–	–	–	–	–	–	–	–	–	0.7	–	–	–	–	–	–	–	–	−0.7058449	1.49071
2.0	1.0	0.1	0.1	0.5	1.0	0.2	0.2	2.5	0.5	6.5	0.5	0.1	6.0	1.0	0.1	0.1	0.1	−0.7102462	1.49006
–	–	–	–	–	–	–	–	–	–	6.6	–	–	–	–	–	–	–	−0.7105847	1.49031
–	–	–	–	–	–	–	–	–	–	6.7	–	–	–	–	–	–	–	−0.7109093	1.49056
2.0	1.0	0.1	0.1	0.5	1.0	0.2	0.2	2.5	0.5	6.5	0.3	0.1	6.0	1.0	0.1	0.1	0.1	−0.7172807	1.48684
–	–	–	–	–	–	–	–	–	–	–	0.4	–	–	–	–	–	–	−0.7137456	1.48846
–	–	–	–	–	–	–	–	–	–	–	0.5	–	–	–	–	–	–	−0.7102462	1.49006
2.0	1.0	0.1	0.1	0.5	1.0	0.2	0.2	2.5	0.5	6.5	0.5	0.2	0.1	6.0	1.0	0.1	0.1	−0.6960295	1.49543
–	–	–	–	–	–	–	–	–	–	–	–	0.4	–	–	–	–	–	−0.6622033	1.50456
–	–	–	–	–	–	–	–	–	–	–	–	0.6	–	–	–	–	–	−0.5980091	1.52416
2.0	1.0	0.1	0.1	0.5	1.0	0.2	0.2	2.5	0.5	6.5	0.5	0.1	6.0	1.0	0.1	0.1	0.1	−0.7102462	1.49006
–	–	–	–	–	–	–	–	–	–	–	–	–	6.5	–	–	–	–	−0.708567	1.48894
–	–	–	–	–	–	–	–	–	–	–	–	–	7.0	–	–	–	–	−0.7068303	1.48797
2.0	1.0	0.1	0.1	0.5	1.0	0.2	0.2	2.5	0.5	6.5	0.5	0.1	6.0	1.0	0.1	0.1	0.1	−0.7102462	1.49006
–	–	–	–	–	–	–	–	–	–	–	–	–	–	1.5	–	–	–	−0.7216742	1.49142
–	–	–	–	–	–	–	–	–	–	–	–	–	–	2.0	–	–	–	−0.7306224	1.49198
2.0	1.0	0.1	0.1	0.5	1.0	0.2	0.2	2.5	0.5	6.5	0.5	0.1	6.0	1.0	0.1	0.1	0.1	−0.7102462	1.49006
–	–	–	–	–	–	–	–	–	–	–	–	–	–	–	0.3	–	–	−0.4709938	1.48784
–	–	–	–	–	–	–	–	–	–	–	–	–	–	–	0.5	–	–	−0.3535851	1.4871
2.0	1.0	0.1	0.1	0.5	1.0	0.2	0.2	2.5	0.5	6.5	0.5	0.1	6.0	1.0	0.1	0.1	0.1	−0.7102462	1.49006
–	–	–	–	–	–	–	–	–	–	–	–	–	–	–	–	0.3	–	−0.715691	1.47083
–	–	–	–	–	–	–	–	–	–	–	–	–	–	–	–	0.5	–	−0.7201997	1.45331
2.0	1.0	0.1	0.1	0.5	1.0	0.2	0.2	2.5	0.5	6.5	0.5	0.1	6.0	1.0	0.1	0.1	0.1	−0.7102462	1.49006
–	–	–	–	–	–	–	–	–	–	–	–	–	–	–	–	–	0.3	−0.7223856	1.4877
–	–	–	–	–	–	–	–	–	–	–	–	–	–	–	–	–	0.5	−0.7320851	1.48572

**Table 2:** The numerical calculations computed for the Nusselt number ${-\theta }^{\prime }\left(0\right)$ and Sherwood number ${-\varphi }^{\prime }\left(0\right)$ by setting m=0.5 fixed.

n	K	β	$\beta_{1}$	γ	λ	ω	ε	Nb	Nt	Pr	ccc	δ	Nr	Sc	$\delta_{1}$	$\delta_{2}$	$\delta_{3}$	${-\theta }^{\prime }\left(0\right)$	${-\varphi }^{\prime }\left(0\right)$
2.0	1.0	0.1	0.1	0.5	0.1	0.2	0.2	2.5	0.5	6.5	0.5	0.1	6.0	1.0	0.1	0.1	0.1	−0.255494	0.542038
3.0	–	–	–	–	–	–	–	–	–	–	–	–	–	–	–	–	–	−0.214219	0.535947
4.0	–	–	–	–	–	–	–	–	–	–	–	–	–	–	–	–	–	−0.189264	0.532516
2.0	1.0	0.1	0.1	0.5	0.1	0.2	0.2	2.5	0.5	6.5	0.5	0.1	6.0	1.0	0.1	0.1	0.1	−0.214219	0.535947
–	2.0	–	–	–	–	–	–	–	–	–	–	–	–	–	–	–	–	−0.297645	0.546841
–	3.0	–	–	–	–	–	–	–	–	–	–	–	–	–	–	–	–	−0.307756	0.549038
2.0	1.0	0.2	0.1	0.5	2.0	0.2	0.2	2.5	0.5	6.5	0.5	0.1	6.0	1.0	0.1	0.1	0.1	0.995294	0.630767
–	–	0.3	–	–	–	–	–	–	–	–	–	–	–	–	–	–	–	0.893102	0.625189
–	–	0.4	–	–	–	–	–	–	–	–	–	–	–	–	–	–	–	0.817999	0.615088
2.0	1.0	0.1	0.1	0.5	0.1	0.2	0.2	2.5	0.5	6.5	0.5	0.1	6.0	1.0	0.1	0.1	0.1	−0.214219	0.535947
–	–	–	0.2	–	–	–	–	–	–	–	–	–	–	–	–	–	–	−0.19743	0.54667
–	–	–	0.3	–	–	–	–	–	–	–	–	–	–	–	–	–	–	−0.140714	0.551129
2.0	1.0	0.1	0.1	0.6	0.1	0.2	0.2	2.5	0.5	6.5	0.5	0.1	6.0	1.0	0.1	0.1	0.1	−0.266432	0.541239
–	–	–	–	0.7	–	–	–	–	–	–	–	–	–	–	–	–	–	−0.274488	0.540655
–	–	–	–	0.8	–	–	–	–	–	–	–	–	–	–	–	–	–	−0.28063	0.540211
2.0	1.0	0.1	0.1	0.5	1.0	0.2	0.2	2.5	0.5	6.5	0.5	0.1	6.0	1.0	0.1	0.1	0.1	0.596467	0.620215
–	–	–	–	–	1.5	–	–	–	–	–	–	–	–	–	–	–	–	0.814714	0.647616
–	–	–	–	–	2.0	–	–	–	–	–	–	–	–	–	–	–	–	0.960849	0.671059
2.0	1.0	0.1	0.1	0.5	1.0	0.2	0.2	2.5	0.5	6.5	0.5	0.1	6.0	1.0	0.1	0.1	0.1	0.596467	0.620215
–	–	–	–	–	–	0.4	–	–	–	–	–	–	–	–	–	–	–	0.648774	0.625754
–	–	–	–	–	–	0.6	–	–	–	–	–	–	–	–	–	–	–	0.681702	0.631469
2.0	1.0	0.1	0.1	0.5	1.0	0.2	0.2	2.5	0.5	6.5	0.5	0.1	6.0	1.0	0.1	0.1	0.1	0.596467	0.620215
–	–	–	–	–	–	–	0.4	–	–	–	–	–	–	–	–	–	–	0.610099	0.620039
–	–	–	–	–	–	–	0.6	–	–	–	–	–	–	–	–	–	–	0.623129	0.619875
2.0	1.0	0.1	0.1	0.5	1.0	0.2	0.2	2.5	0.5	6.5	0.5	0.1	6.0	1.0	0.1	0.1	0.1	0.596467	0.620215
–	–	–	–	–	–	–	–	2.6	–	–	–	–	–	–	–	–	–	0.557008	0.6207
–	–	–	–	–	–	–	–	2.7	–	–	–	–	–	–	–	–	–	0.51209	0.621314
2.0	1.0	0.1	0.1	0.5	1.0	0.2	0.2	2.5	0.5	6.5	0.5	0.1	6.0	1.0	0.1	0.1	0.1	0.596467	0.620215
–	–	–	–	–	–	–	–	–	0.6	–	–	–	–	–	–	–	–	0.580267	0.623085
–	–	–	–	–	–	–	–	–	0.7	–	–	–	–	–	–	–	–	0.557258	0.626221
2.0	1.0	0.1	0.1	0.5	1.0	0.2	0.2	2.5	0.5	6.5	0.5	0.1	6.0	1.0	0.1	0.1	0.1	0.596467	0.620215
–	–	–	–	–	–	–	–	–	–	6.6	–	–	–	–	–	–	–	0.581387	0.620359
–	–	–	–	–	–	–	–	–	–	6.7	–	–	–	–	–	–	–	0.566328	0.620506
0.1	6.0	1.0	0.1	0.1	0.1	0.1	6.0	1.0	0.1	0.1	0.3	0.1	6.0	1.0	0.1	0.1	0.1	0.789889	0.615534
–	–	–	–	–	–	–	–	–	–	–	0.4	–	–	–	–	–	–	0.692376	0.617895
–	–	–	–	–	–	–	–	–	–	–	0.5	–	–	–	–	–	–	0.596467	0.620215
2.0	1.0	4.0	0.3	0.2	1.0	0.2	0.2	2.5	0.5	6.5	0.5	0.7	0.1	6.0	1.0	0.1	0.1	−2.13426	0.651445
–	–	–	–	–	–	–	–	–	–	–	–	1.0	–	–	–	–	–	−1.27322	0.497982
–	–	–	–	–	–	–	–	–	–	–	–	2.0	–	–	–	–	–	−0.677065	0.440631
2.0	1.0	0.1	0.1	0.5	1.0	0.2	0.2	2.5	0.5	6.5	0.5	0.1	6.0	1.0	0.1	0.1	0.1	0.596467	0.620215
–	–	–	–	–	–	–	–	–	–	–	–	–	6.5	–	–	–	–	0.712513	0.619624
–	–	–	–	–	–	–	–	–	–	–	–	–	7.0	–	–	–	–	0.828584	0.619175
2.0	1.0	0.1	0.1	0.5	1.0	0.2	0.2	2.5	0.5	6.5	0.5	0.1	6.0	1.0	0.1	0.1	0.1	0.596467	0.620215
–	–	–	–	–	–	–	–	–	–	–	–	–	–	1.5	–	–	–	0.514656	0.767486
–	–	–	–	–	–	–	–	–	–	–	–	–	–	2.0	–	–	–	0.481088	0.886847
2.0	1.0	0.1	0.1	0.5	1.0	0.2	0.2	2.5	0.5	6.5	0.5	0.1	6.0	1.0	0.1	0.1	0.1	0.596467	0.620215
–	–	–	–	–	–	–	–	–	–	–	–	–	–	–	0.3	–	–	0.729662	0.596215
–	–	–	–	–	–	–	–	–	–	–	–	–	–	–	0.5	–	–	0.773871	0.584323
2.0	1.0	0.1	0.1	0.5	1.0	0.2	0.2	2.5	0.5	6.5	0.5	0.1	6.0	1.0	0.1	0.1	0.1	0.596467	0.620215
–	–	–	–	–	–	–	–	–	–	–	–	–	–	–	–	0.3	–	0.583439	0.6193
–	–	–	–	–	–	–	–	–	–	–	–	–	–	–	–	0.5	–	0.560294	0.618758
2.0	1.0	0.1	0.1	0.5	1.0	0.2	0.2	2.5	0.5	6.5	0.5	0.1	6.0	1.0	0.1	0.1	0.1	0.596467	0.620215
–	–	–	–	–	–	–	–	–	–	–	–	–	–	–	–	–	0.3	0.737779	0.549385
–	–	–	–	–	–	–	–	–	–	–	–	–	–	–	–	–	0.5	0.857055	0.493204

The heat transfer improved while reduced the mass transfer due to heightening values of n. The shear thickness increased which increased the heat transfer due to larger values of n while mass transfer reduced to shear thickness enlarging. The values of K enlarged which improved the mass and heat transfer at surface. Because, the fluid rotation increased which increased mass and heat transfer at surface. Heat transfer reduced while mass transfer declined for higher values of β. The increment in β which improved the shear thinning as well as transfer of heat reduced and transfer of mass reduced at surface. Heat transfer improved while mass transfer improved for higher values of $\beta_{1}$. The Laurent forces developed the resistance between surface and fluid due to reduction in the momentum thickness. The increment in $\beta_{1}$ which improved the shear thinning as well as transfer of heat improved and transfer of mass improved at surface. It is manifested that both transfer of mass and heat are decayed by varying γ to bigger values. Physically, the gap between surface and magnetic field declined exponentially which reduced the momentum thickness ultimately, both transfer of mass and heat are decayed at surface. It is manifested that transfer of mass and heat are enlarged by varying λ to bigger values. The buoyancy force heightened the transfer of mass and heat between surface and fluid interaction point. It is manifested that both transfer of mass and heat are enlarged by varying ω to bigger values. It is displayed that transfer of mass decayed and transfer of heat heightened by varying ε to bigger values. Thermal conductivity improved which improved the heat transfer phenomena, so transfer of heat at surface become larger while also reduced the transfer of mass. The transfer of heat decayed due to increment in Nb while increment in Nb which heightened the transfer of mass. The transfer of heat decayed due to increment in Nt while increment in Nt which heightened the transfer of mass. The transfer of mass values heightened and transfer of heat values reduced due to enlarging values of Pr. The diffusion of momentum enlarged due to heightening the values of Pr as well as reduced the transfer of heat and increment in transfer of mass. The transfer of heat is decreased whereas transfer of mass is increased, when values of Ec increased. If the values of Ec boosted up which heightened the kinetic energy as well as increment in momentum. Due to increment in momentum, the transfer of heat is decreased whereas transfer of mass is increased at surface. Transfer of mass and heat is increased, when values of δ increased. If the values of δ boosted up which heightened the kinetic energy as well as increment in momentum. Due to increment in momentum, the transfer of mass and heat is increased at surface. Transfer of mass and heat is decayed, when values of Nr increased. If the values of Nr boosted up which heightened the kinetic energy as well as increment in momentum. Due to increment in momentum, the transfer of mass and heat is increased at surface. The transfer of heat is decreased whereas transfer of mass is increased, when values of Sc increased. The reason for the rise in the Sc values reduces the molecular diffusivity and this further reduces the concentration gradient. Which boosted up the mass transfer and reduced heat transfer phenomena. The heighten values of $\delta_{1}$ which reduced the transfer of mass and heat at surface. As the slip factor boosted up which reduced the transfer of mass and heat between fluid and surface. The increment in $\delta_{2\;}$ which reduced the heat while heightened the mass transfer. The thermal slip impacts implemented on the mass and heat transfer which declined heat which boosted up mass transfer. The increment in $\delta_{3\;}$ which reduced the transfer of mass and heat. Comparison present work with Waqas et al. [34] and Cortell [35] for different values of n is reported in Table 3. The results is found to be good agreement with Waqas et al. [34] and Cortell [35]. Table 4 is provided the methods comparison ND-solver and bvp4c. It is noted that these results are approximately same.

**Table 3:** Comparison present work with Waqas et al. [34] and Cortell [35] for different values of n.

n	Present results	Waqas et al. [34]	Cortell [35]
0.000	0.62735020	0.6276	0.627547
0.200	0.76689401	0.7669	0.766758
0.500	0.88957644	0.8895	0.889477
0.750	0.95395665	0.9539	0.953786
1.000	1.00000000	1.0000	1.000000
1.500	1.06160208	1.0616	1.061587
3.000	1.14859409	1.1486	1.148588
7.000	1.21685388	1.2169	1.216847
10.00	1.23487918	1.2349	1.234875
20.00	1.25742989	1.2575	1.257418
100.0	1.27678157	1.2768	1.276768

**Table 4:** Comparative methods of Bvp4c and ND-solver for different values of parameters.

			Bvp4c method		ND-solver method	
K	β	$\beta_{1}$	${C_{f}Re}_{e}^{1/2}$	${C_{m}R}_{e}^{1/2}$	${C_{f}Re}_{e}^{1/2}$	${C_{m}R}_{e}^{1/2}$
1.0	0.1	0.1	−1.376887	1.49722	−1.375784	1.496873
2.0	–	–	−1.634629	1.99180	−1.633531	1.990953
3.0	–	–	−1.849252	2.48631	−1.848791	2.485931
1.0	2.0	0.3	−1.64048	1.96015	−1.640375	1.960093
-	3.0	–	−1.672719	1.73266	−1.671677	1.731689
-	4.0	–	−1.835320	1.68360	−1.834721	1.682783
1.0	0.1	0.1	−1.376887	1.49722	−1.375624	1.496712
-	–	0.2	−1.350265	1.49648	−1.350184	1.495862
-	–	0.3	−1.323270	1.49579	−1.322571	1.494713

Fig. 2, Fig. 3, Fig. 4, Fig. 5 reveal the effect of $\beta_{1}$, K, λ and ω parameters on $f^{\prime }\left(\eta \right)$. The velocity heightened due to increment in the $\beta_{1}$ which is reported in Fig. 2. The Laurent forces developed the resistance between surface and fluid due to reduction in the momentum thickness ultimately, velocity increased. The variation of $f^{\prime }\left(\eta \right)$ and ω reported in Fig. 3. Velocity curves reported boosted up due to higher values of ω. The buoyancy force heightened which improved the kinetic energy ultimately, improved the velocity of fluid. Curves of velocity improved due to heighten values of K that reported in Fig. 4. Physically, it is evident that larger values of K agree to the lower viscosity of the fluid. That is why fluid becomes thinner which results to enhance velocity. The rotation of the particle increased which boosted up the kinetic energy of the particle as well as velocity improved. Influence of λ on velocity reported in Fig. 5. Velocity curves heightened due to enlarging values of λ. The mixed convection parameter λ illustrates the relative impacts of the thermal buoyancy force to the viscous hydrodynamic force in the system. The increment of this parameter specify the additional buoyancy forces, which improves the motion of fluid. Fig. 6, Fig. 7 reported the variation of β and K on h(η). The impact of β on h(η) is reported in Fig. 6. The curves of h(η) boosted up due to enlarging values of β. Physically, the heighten values of β which leads to reduce the momentum thickness ultimately, heightened the micropolar profile. The impact of K on h(η) is reported in Fig. 7. The curves of h(η) boosted up due to enlarging values of K. The micropolar parameter boosted up which boosted up the rotation of micropolar profile. The influence of ε, K, Nb, Nt, Nr and Pr on the temperature which reported in Fig. 8, Fig. 9, Fig. 10, Fig. 11, Fig. 12, Fig. 13. Influence of ϵ on temperature is reported in Fig. 8. Temperature curves heightened by increasing the ϵ. Physically, thermal conductivity of liquid boosted up due to enlarging values of ϵ which improved the temperature. Influence of K on temperature is reported in Fig. 9. Temperature curves decayed by increasing the K. The rotation of fluid particle heightened due to larger values of K as well as temperature reduced at surface. Fig. 10 stated the inspiration of Nb on the θ(η). The curves of θ(η) enlarged due to higher values of Nb. Fig. 11 stated the inspiration of Nt on the θ(η). The curves of θ(η) enlarged due to higher values of Nt. The thermophoretic force improves the movement of particles and fluid flow is faster away from the sheet surface. Additionally, in relation to higher values of Nb, Brownian motion presents disorderness. The large amounts of heat observed when Nb and Nt both are increased which ultimately leads to a rise in the temperature profiles. θ(η) increased by rising the value of Nr reported in Fig. 12. Physically, when the quantity of heat produced through thermal radiation parameter enhancement, the fluid temperature distribution will rise. θ(η) decreased by rising the value of Pr as shown in Fig. 13. Physically, an enhancement of Prandtl number results the decrease in the thermal conductivity and therefore the thermal boundary layer thickness decreases. The variations in φ(η) consequent to numerous values of $\beta ,\delta_{3},K$ and Sc are sketched in Fig. 14, Fig. 15, Fig. 16, Fig. 17. Fig. 14 indicates that φ(η) increases by increasing β. Because, thermal conductivity of fluid heightened due to higher values of β. Ultimately, concentration profile boosted up. The impact of $\delta_{3}$ on the φ(η) reported in Fig. 15. The curves of φ(η) decayed due to higher values of $\delta_{3}$. As the values of $\delta_{3}$ higher, which declined the concentration curves. φ(η) is shown decline when the values of K augment as plotted in Fig. 16. φ(η) expresses decreasing behavior in case of increment in the values of Sc which is seen in Fig. 17. The physical fact is that Sc represents the ratio of viscosity and mass diffusivity. The reason for the rise in the Sc values reduces the molecular diffusivity and this further reduces the concentration gradient. Due to this reason, φ(η) is reduced.

## 5 Conclusion

The nonlinear vertical stretching Riga sheet is considered to analyze the impact of unsteady micropolar fluid. The temperature-dependent properties under heat generation, radiation, and viscous dissipation have been studied. The thermal, velocity, and concentration slip impacts are taken into account. The governing flow model has been developed under flow assumptions and solved through a numerical scheme. The physical interpretation of flow analysis has been presented through graphs and tabular form. The main points are highlighted below.

• The K increased which increased the couple stress and friction at surface. Because, the fluid rotation increased which increased friction at surface and also increased the couple stress.
• The transfer of mass decayed and transfer of heat heightened by the bigger values ε. Thermal conductivity improved which improved the heat transfer phenomena, so transfer of heat at surface becomes larger while also reducing the transfer of mass.
• The impact of K on the skin friction and couple's stress noted. The K increased which increased the couple stress and friction at surface. Because, the fluid rotation increased which increased friction at surface and also increased the couple stress.
• The both friction and couple stress are increased by varying γ to bigger values. Physically, the gap between surface and magnetic field declined exponentially which reduced the momentum thickness and ultimately, increased the friction at surface.

The presented results will be extended for non-Newtonian model under the micropolar fluid effects.

## Data availability

No data was used for the research described in the article.

## Funding

No funding

## CRediT authorship contribution statement

**Nadeem Abbas:** Writing - review & editing, Writing - original draft, Methodology, Conceptualization. **Mohsin Ali:** Writing - review & editing, Writing - original draft, Software. **Wasfi Shatanawi:** Supervision, Methodology, Investigation. **Fady Hasan:** Validation, Methodology, Formal analysis.

## Declaration of competing interest

The authors declare no conflict of interest.
